# Evaluating inhaled corticosteroids' impact on osteoporosis and fracture risk in COPD patients: a real-world evidence-based systematic review and meta-analysis

**DOI:** 10.3389/fmed.2025.1503475

**Published:** 2025-06-06

**Authors:** Hsiao-Feng Huang, Chin-Wei Hsu, Fei-Ting Hsieh, Kuang-Ming Liao

**Affiliations:** ^1^Department of Pharmacy, Chi Mei Medical Center, Chiali, Taiwan; ^2^Department of Pharmacy, Chi Mei Medical Center, Tainan, Taiwan; ^3^School of Pharmacy, Kaohsiung Medical University, Kaohsiung, Taiwan; ^4^Institute of Clinical Pharmacy and Pharmaceutical Sciences, College of Medicine, National Cheng Kung University, Tainan, Taiwan; ^5^Department of Internal Medicine, Chi Mei Medical Center, Chiali, Taiwan; ^6^Department of Nursing, Min-Hwei Junior College of Health Care Management, Tainan, Taiwan

**Keywords:** chronic obstructive pulmonary disease, inhaled corticosteroids, meta-analysis, systematic review, osteoporosis, fracture

## Abstract

**Background:**

The impact of current inhaled corticosteroid (ICS) therapies on fracture risk in patients with chronic obstructive pulmonary disease (COPD) remains uncertain.

**Objective:**

This study conducts a systematic review and meta-analysis to assess the risk of fractures associated with ICS use over at least 4 years, synthesizing evidence from observational studies conducted in real-world settings among individuals with COPD.

**Methods:**

We systematically searched PubMed, EMBASE, Scopus, and Web of Science from inception to April 21, 2025. Inclusion criteria encompassed studies conducted in COPD patients, evaluating interventions involving ICS-containing treatments compared to alternatives or no ICS use, using cohort or case-control designs, and reporting outcomes related to osteoporosis or fractures. Pooled odds ratios (OR) and hazard ratios (HR) were calculated using random-effects models. Subgroup analyses and meta-regression were performed to explore sources of heterogeneity.

**Results:**

Nine studies (six case-control, three cohort) were included. The pooled OR from case-control studies was 1.03 (95% CI: 0.99–1.08; *I*^2^ = 50%), and the pooled HR from cohort studies was 0.95 (95% CI: 0.67–1.33; *I*^2^ = 86%). Subgroup analyses indicated a potential increased risk in Asian and European populations but not in North America. Meta-regression revealed that higher oral corticosteroids exposure was significantly associated with increased risk (*p* = 0.005, *R*^2^ = 100%).

**Conclusions:**

Although ICS did not significantly impact osteoporosis or fracture risk, these are common comorbidities in COPD patients. Methodological differences, such as study design, outcome definitions, and oral corticosteroids use, may influence result interpretation and contribute to heterogeneity, limiting study comparability.

## Introduction

In individuals with moderate to very severe COPD and frequent exacerbation, a therapy that combines an inhaled corticosteroid (ICS) with a long-acting beta-agonist (LABA) is superior to using either medication by itself. ICS/LABA not only improves respiratory function but also diminishes the frequency of COPD exacerbations. Significant evidence from previous studies show the superiority of combining ICS/LABA over using LABA alone, particularly in patients who have had at least one exacerbation in the preceding year (1–5). For patients with COPD who have experienced previous exacerbations, a Randomized Controlled Trial (RCT) conducted in primary healthcare settings across the United Kingdom revealed that a daily combined therapy of ICS/LABA was linked to a reduction in exacerbation frequency compared to standard treatment, without an increased occurrence of serious adverse effects. The incidence of moderate or severe exacerbations was notably reduced by 8.4% in those receiving ICS/LABA compared to those under usual care (6).

Furthermore, several research findings indicate that the level of blood eosinophils can forecast the effectiveness of adding ICS to ongoing maintenance bronchodilator therapy in averting future exacerbations. The impact of ICS is directly correlated with blood eosinophil levels; at lower eosinophil counts, minimal or no benefits are noted, while the benefits progressively increase with higher eosinophil counts (7–12).

Findings from RCTs on the association between ICS treatment and the risk of reduced bone density and fractures have been inconsistent, possibly because of variations in study methodologies or differences among the ICS formulations (13–17).

Previous meta-analyses have faced limitations, including short follow-up durations and incomplete coverage of all ICS treatments. Additionally, recent observational studies, drawing on national databases from various countries (18–20), have yielded inconsistent findings. As a result, the effects of the ICS treatments currently in use on fracture risk in COPD patients are still not well-defined. Our study systematically reviews the risk of fractures associated with at least 4 years of ICS use, examining evidence from observational studies conducted in real-world settings among individuals with COPD.

## Methods

### Search strategy

This systematic review was conducted according to the PRISMA 2020 guidelines and registered on PROSPERO (CRD42024520403). Four databases (PubMed, EMBASE, Scopus, Web of Science) were searched from their inception to April 21, 2025. The following medical subject headings terms were used: “Pulmonary Disease, Chronic Obstructive,” “Administration, Inhalation,” “Nebulizers and Vaporizers,” “Adrenal Cortex Hormones,” “Budesonide,” “Fluticasone,” “Beclomethasone,” “Mometasone Furoate,” “Osteoporosis,” “Fractures, Bone,” “bone density,” “skeletal health,” and “bone health.” No language restrictions were imposed. Detailed search strategies are presented in Supplementary Table S1.

### Study selection

Inclusion criteria for studies were as follows: (i) patients diagnosed with COPD; (ii) use of ICS-containing treatments for intervention; (iii) comparison with alternative treatments or no ICS usage; (iv) design as a cohort or case-control study; and (v) reported outcomes of osteoporosis or fractures. Exclusion criteria included: (i) studies not reporting relevant outcomes, and (ii) studies on non-human subjects. Additionally, conference abstracts, commentaries, narrative reviews, and case reports were not considered.

This review was structured based on the PICO framework: Population (adults with COPD), Intervention (ICS use), Comparison (no ICS), and Outcomes (osteoporosis or fracture).

Two investigators (SFH and FTH) independently screened the titles and abstracts of the records collected using the aforementioned search strategies to identify and assess potentially eligible studies. Disagreements were resolved by a third investigator (KML). Full-text copies of potentially relevant articles were obtained and reviewed for eligibility.

### Data extraction

Data were independently extracted by two investigators (CWH and KML) using a standardized electronic form. Extracted data included: study characteristics (author, year, design, country), population demographics (sample size, sex, age), definitions of COPD and outcomes, ICS types and doses, oral corticosteroid (OCS) and bisphosphonate use, and reported effect estimates with confidence intervals. Disagreements were resolved by a third investigator (SFH).

### Quality assessment

Two investigators (CWH and KML) independently assessed the quality for each of the included studies by using the Newcastle–Ottawa Scale (NOS). Disagreements were resolved through discussion and consensus with a third investigator (SFH).

### Statistical analysis

Meta-analysis was conducted using Review Manager (RevMan, version 5.4; Nordic Cochrane Center, Copenhagen, Denmark) and R software (version 4.5.0) for meta-regression analysis. For case-control studies, odds ratios (ORs) with 95% confidence intervals (CIs) were pooled using the inverse-variance random-effects model. Hazard ratios (HRs) from cohort studies were likewise combined using the inverse-variance random-effects model. Heterogeneity was assessed using Cochran's Q test and quantified with the *I*^2^ statistic, with *I*^2^ values interpreted as follows: ≤25% (low), 26–74% (moderate), and ≥75% (high heterogeneity). Subgroup analysis was conducted to explore potential sources of heterogeneity based on (1) outcome definitions (e.g., osteoporosis, any fracture, specific fracture types) and (2) geographic region. Furthermore, meta-regression analysis was performed to examine whether study-level OCS or bisphosphonate exposure influenced the effect estimates. To assess the robustness of the pooled effect estimates and identify influential studies, we conducted a leave-one-out sensitivity analysis.

## Results

### Search results

**Figure 1:**
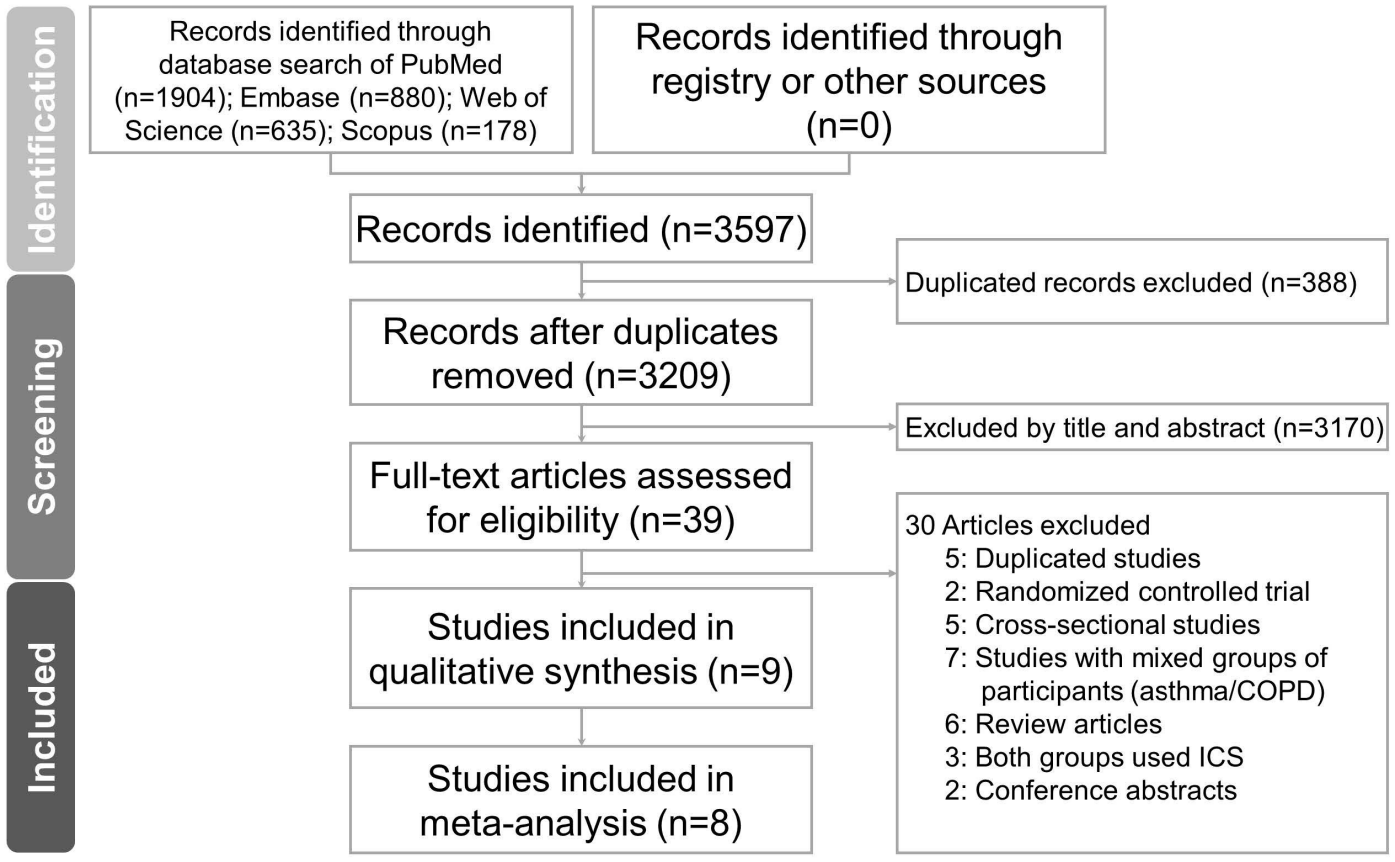
Study selection process of the systematic review and meta-analysis.

A total of 3,597 records were identified through database searching: PubMed (*n* = 1,904), Embase (*n* = 880), Web of Science (*n* = 635), and Scopus (*n* = 178). No additional records were retrieved through trial registries or other sources. After removing 388 duplicates, 3,209 records remained for title and abstract screening. Of these, 3,170 records were excluded due to irrelevance. A total of 39 full-text articles were assessed for eligibility. Following full-text review, 9 studies (18–26) met the inclusion criteria and were included in the final analysis. These comprised 6 case-control studies and 3 cohort studies, as shown in Figure 1.

### Study characteristics

The included studies (18–26) were published between 2004 and 2021 and conducted in Taiwan, the United Kingdom, the United States, Canada, and Sweden. COPD was diagnosed using ICD-9 or ICD-10 codes across all studies. The type of ICS investigated varied and included fluticasone, budesonide, beclomethasone, and fixed-dose combinations (Tables 1, 2). The average patient age ranged from 52.3 to 74.8 years, and the proportion of male participants varied significantly between studies. OCS and bisphosphonate usage rates also differed, ranging from 13.4% to 60.8% and 1.6% to 12.7%, respectively. Study quality, assessed using the NOS, was generally high, with most studies scoring 8 or 9 stars (Tables 3, 4).

**Table 1:** Characteristics of case-control studies of inhaled corticosteroids (ICS) and fractures or osteoporosis.

**Study**	**Data source**	**Study period**	**COPD criteria**	**Type of ICS**	**Patient number**	**Male (%)**	**Mean age (yr)**	**OCS use (%)**	**Bisphosphonates use (%)**
**Osteoporosis**									
Chiu et al. (20)	Taiwan; NHIRD	2003–2016	Newly diagnosed COPD patients, according to ICD-9/10 codes, aged ≥ 40 years	All	Total: 232,192 Case: 58,048 Control: 174,144	Total: 51.24 Case: 51.24 Control: 51.24	Total: 66.3 Case: 66.2 Control: 66.3	Total: 37.1 Case: 38.4 Control: 36.7	NA
**Any fracture**									
Pujades-Rodríguez et al. (21)	UK; The Health Improvement Network	1998–2005	COPD ≥ 40 years	BDP, FP, BDS	Total: 5,833 Case: 1,235 Control: 4,598	Total: 41.3 Case: 40 Control: 41.6	Total: 67.9 Case: 69.3 Control: 67.6	Total: 56.7 Case: 59.6 Control: 55.9	Total: 8.8 Case: 12.6 Control: 7.7
**Hip or upper extremity fracture**									
Gonzalez et al. (18)	Canada; RAMQ	1990–2007	Newly treated for COPD, aged > 55 years	All	Total: 403,874 Case: 19,396; Control: 384,478	Total: 27.8 Case: 27.9 Control: 27.8	Total: 74.8 Case: 74.9 Control: 74.8	Total: 13.4 Case: 14.5 Control: 13.3	Total: 4.9 Case: 6.6 Control: 4.8
**Non-vertebral fracture**									
Lee and Weiss (22)	US; American Veterans Affairs patients	1998–2002	New diagnosis of COPD according to ICD-9 codes	All	Total: 8,525 Case: 1,708 Control: 6,817	Total: 94.5 Case: 94.4 Control: 94.6	Total: 62.7	Total: 17.7 Case: 21.3 Control: 16.8	Total: 1.7 Case: 2.4 Control: 1.6
Johannes et al. (23)	US; United Healthcare database	1997–2001	Physician's diagnosis of COPD according to ICD-9 codes, aged ≥40 years	All	Total: 18,942 Cases: 1,722; Control: 17,220	Total: 40.0 Case: 29.4 Control: 41.1	Total: 52.3 Case: 52.9 Control: 52.2	Total: 26.5 Case: 26.4 Control: 26.5	Total: 1.6 Case: 3.0 Control: 1.5
Miller et al. (24)	UK; GPRD	2003–2006	Physician-diagnosed COPD, aged ≥45 years	FSC, other ICS	Total: 5,272 Case: 1,523 Control: 3,749	Total: 37.4 Case: 36.6 Control: 37.7	NA	Total: 39.4 Case: 38.7 Control: 39.7	Total: 12.7 Case: 15.2 Control: 11.6

BDS, budesonide; BDP, beclometasone dipropionate; FP, fluticasone propionate; FSC, fluticasone propionate/salmeterol fixed-dose combination; GPRD, General Practice Research Database; ICS, Inhaled corticosteroid; ICD-9/10, International Classification of Diseases, Nineth/Tenth Revision; NA, Not available; NHIRD, National Health Insurance Research Database; OCS, oral corticosteroid; RAMQ, Régie de l'assurance maladie du Québec (Quebec health-care databases); yr: year.

**Table 2:** Characteristics of cohort studies of inhaled corticosteroids (ICS) and fractures or osteoporosis.

**Study**	**Liu et al. (25)**	**Price et al. (26)**	**Janson et al. (21)**
Location; data source	Taiwan; NHIRD	UK; CPRD; and OPCRD	Sweden; National Patient Register, National Prescription Register and Cause of Death Register
Study period	1996–2011	1990–2015	2000–2014
COPD criteria	Newly diagnosed female COPD according to ICD-9 codes, ≥40 years	Physician-diagnosed COPD, aged ≥40 years	Physician's diagnosis of COPD according to ICD-10 codes, aged ≥40 years
Type of ICS	All	All	Budesonide, fluticasone propionate
Patient number	ICS user: 812 No ICS: 9,911	ICS user: 12,619; LABD user: 7,279	High dose ICS (<640 μg/day): 580 Low dose ICS (≥640μg/day):4,256 No ICS: 4,815
Male (%)	0	ICS user: 62.5 LABD user:62.1	49
Mean age, years	NA	ICS user: 67.7 LABD user: 67.9	69.5
Oral steroid use (%)	60.8	ICS user: 22.7 LABD user: 19.2	50.1
Statistical analysis	Cox proportional hazard regression	Propensity score matching Cox proportional hazards regression	Multivariate regression
Outcomes (95% CI)	ICS user vs. No ICS Osteoporosis: HR: 0.80 (0.68–0.92)	ICS user vs. LABD user Osteoporosis: HR: 1.13 (0.93–1.39)	Low dose ICS vs. No ICS All fractures: RR: 1.19 (1.05–1.21) Fractures typically related to osteoporosis: RR: 1.13 (1.02–1.17) Recorded diagnosis of osteoporosis: RR: 1.28 (1.22–1.33) Prescriptions of drugs for osteoporosis: RR: 1.43 (1.24–1.56) Any osteoporosis-related event: RR: 1.27 (1.13–1.56) High dose ICS vs. No ICS All fractures: RR 1.26 (1.23–1.39) Fractures typically related to osteoporosis: RR: 1.16 (1.05–1.24) Recorded diagnosis of osteoporosis: RR: 1.41 (1.26–1.53) Prescriptions of drugs for osteoporosis: RR: 2.31 (2.12–2.50) Any osteoporosis-related event: RR: 1.52 (1.24–1.82)

CPRD, Clinical Practice Research Datalink; HR, hazard ratio; ICS, Inhaled corticosteroid; ICD-9/10, International Classification of Diseases, Nineth/Tenth Revision; LABD, long-acting bronchodilator; NA, Not available; NHIRD, National Health Insurance Research Database; OPCRD, Optimum Patient Care Research Database; RAMQ, Régie de l'assurance maladie du Québec (Quebec health-care databases); RR, rate ratio.

**Table 3:** Newcastle–Ottawa quality assessment scale: case-control studies.

**Study**	**Case definition**	**Representativeness of cases**	**Selection of controls**	**Definition of controls**	**Comparability of cases and controls**	**Ascertainment of exposure**	**Same method for cases and controls**	**Non-response rate**	**Total stars**
Chiu et al. (20)	1	1	1	1	2	1	1	1	9
Gonzalez et al. (20)	1	1	1	1	2	1	1	1	9
Johannes et al. (23)	1	1	1	1	2	1	1	1	9
Lee and Weiss (22)	1	0	1	1	2	1	1	1	8
Pujades-Rodriguez (21)	1	1	1	1	2	1	1	1	9
Miller et al. (24)	1	1	1	1	2	1	1	1	9

**Table 4:** Newcastle–Ottawa quality assessment scale: cohort studies.

**Study**	**Representativeness of exposed cohort**	**Selection of non-exposed cohort**	**Ascertainment of exposure**	**Demonstration of outcome not present at start**	**Comparability of cohorts**	**Assessment of outcome**	**Follow-up length**	**Adequacy of follow up**	**Total stars**
Janson et al. (19)	1	1	1	1	2	1	1	1	9
Liu et al. (25)	0	1	1	1	2	1	1	1	8
Price et al. (26)	1	1	1	1	2	1	1	1	9

### Meta-analysis of case-control studies

**Figure 2:**
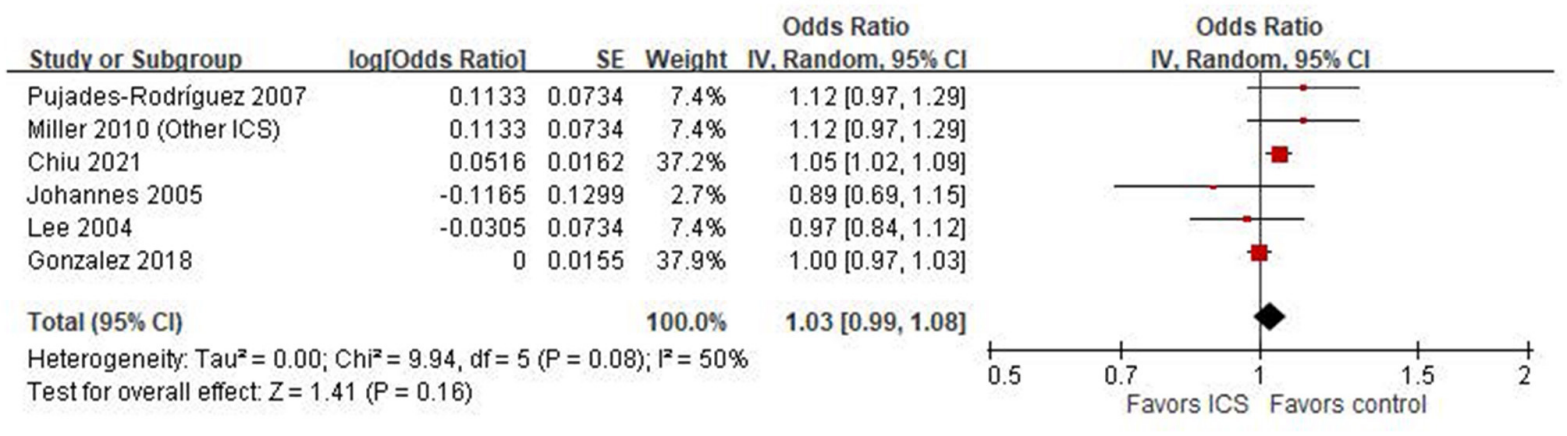
Forest plot of case-control studies examining the association between inhaled corticosteroids (ICS) and the risk of fractures or osteoporosis.

Six case-control studies (18, 20–24) were included in the meta-analysis assessing the association between ICS use and the risk of osteoporosis or fractures. The pooled odds ratio (OR) was 1.03 (95% CI: 0.99–1.08; *P* = 0.16), indicating a non-significant association between ICS exposure and osteoporosis or fractures risk in COPD patients (Figure 2). Heterogeneity was moderate (*I*^2^ = 50%).

### Meta-analysis of cohort studies

**Figure 3:**
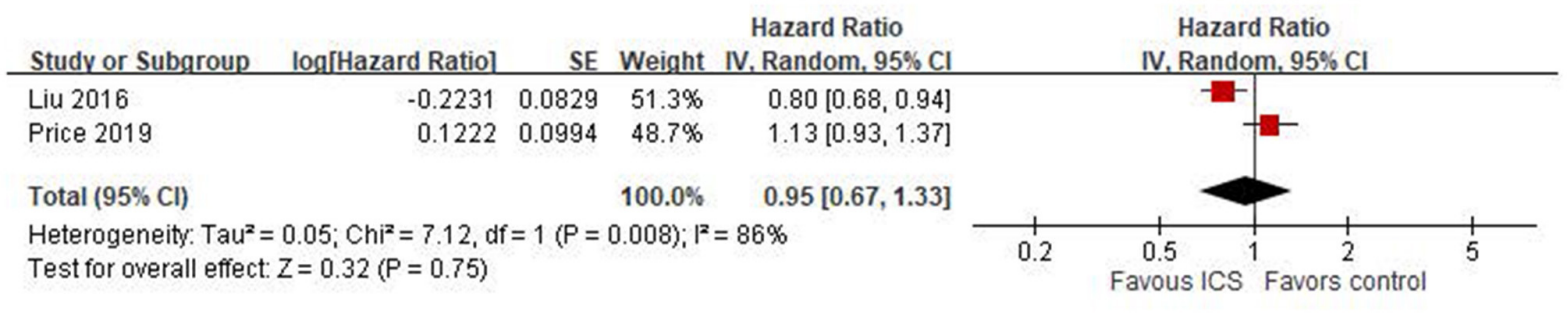
Forest plot of cohort studies using Cox proportional hazard regression assessing ICS-related fracture or osteoporosis risk.

Two cohort studies (25, 26) provided HRs examining the association between ICS use and the risk of osteoporosis or fractures. The pooled HR was 0.95 (95% CI: 0.67–1.33; *P* = 0.75), indicating no significant association between ICS use and osteoporosis or fractures outcomes (Figure 3). However, heterogeneity was high (*I*^2^ = 86%, *P* = 0.008), suggesting substantial variability between studies.

### Subgroup analysis by outcome definition

**Figure 4:**
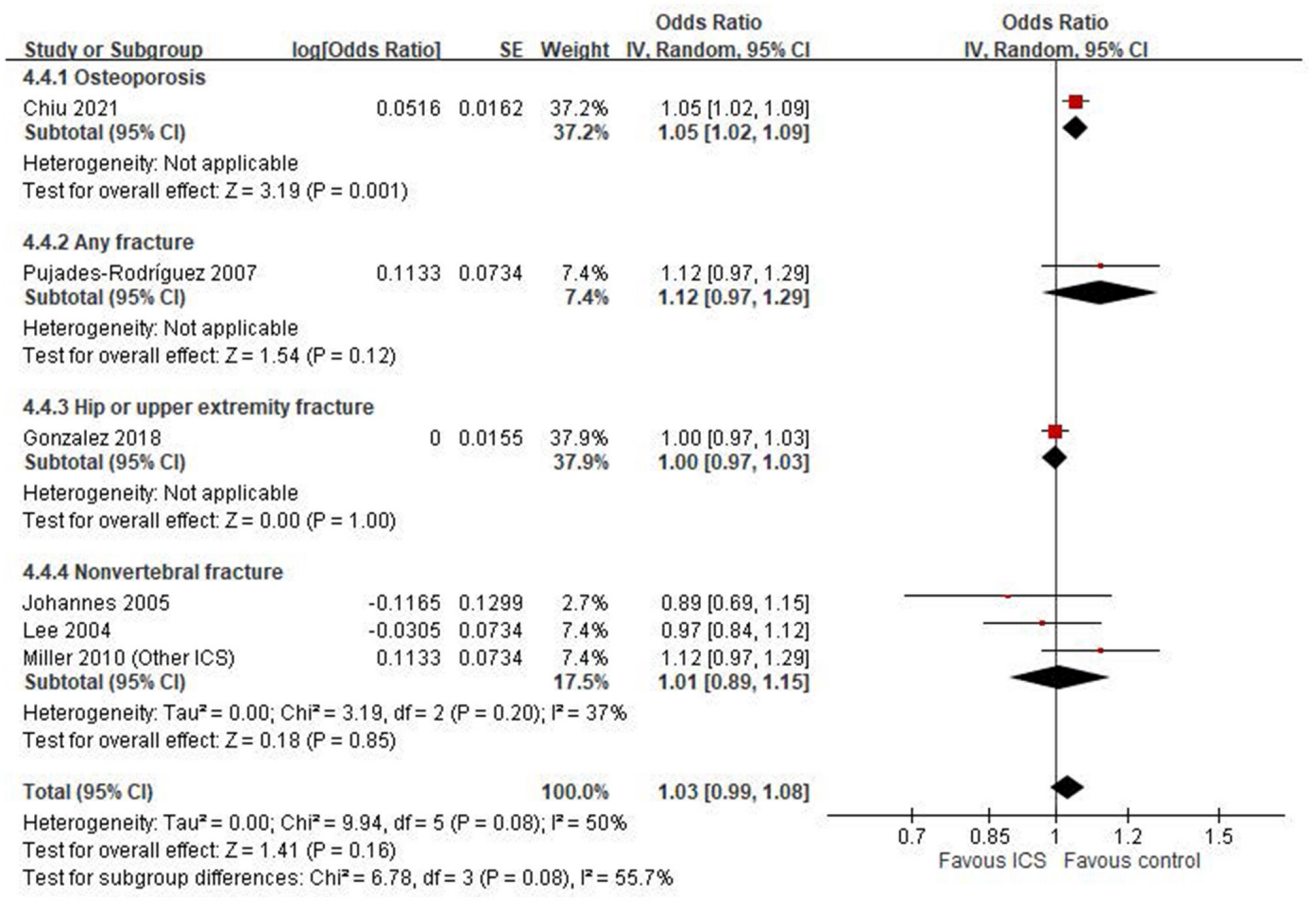
Subgroup analysis by outcome definition: osteoporosis, any fracture, hip or upper extremity fracture and non-vertebral fracture.

We conducted an exploratory subgroup analysis to examine the association between ICS use and specific skeletal outcomes (Figure 4). Among the included studies, only one reported an outcome explicitly defined as osteoporosis (20), which showed a statistically significant association with ICS use (OR: 1.05, 95% CI: 1.02–1.09, *P* = 0.001). For other fracture-related outcomes, results were inconsistent across individual studies. One study (21) reported an increased risk of any fracture (OR: 1.12, 95% CI: 0.97–1.29), while another (18) found a null association for hip or upper extremity fracture (OR: 1.00, 95% CI: 0.97–1.03). For non-vertebral fractures, a pooled estimate from three studies (22–24) yielded an OR of 1.01 (95% CI: 0.89–1.15, *P* = 0.85), with moderate heterogeneity (*I*^2^ = 37%).

### Subgroup analysis by geographic region

**Figure 5:**
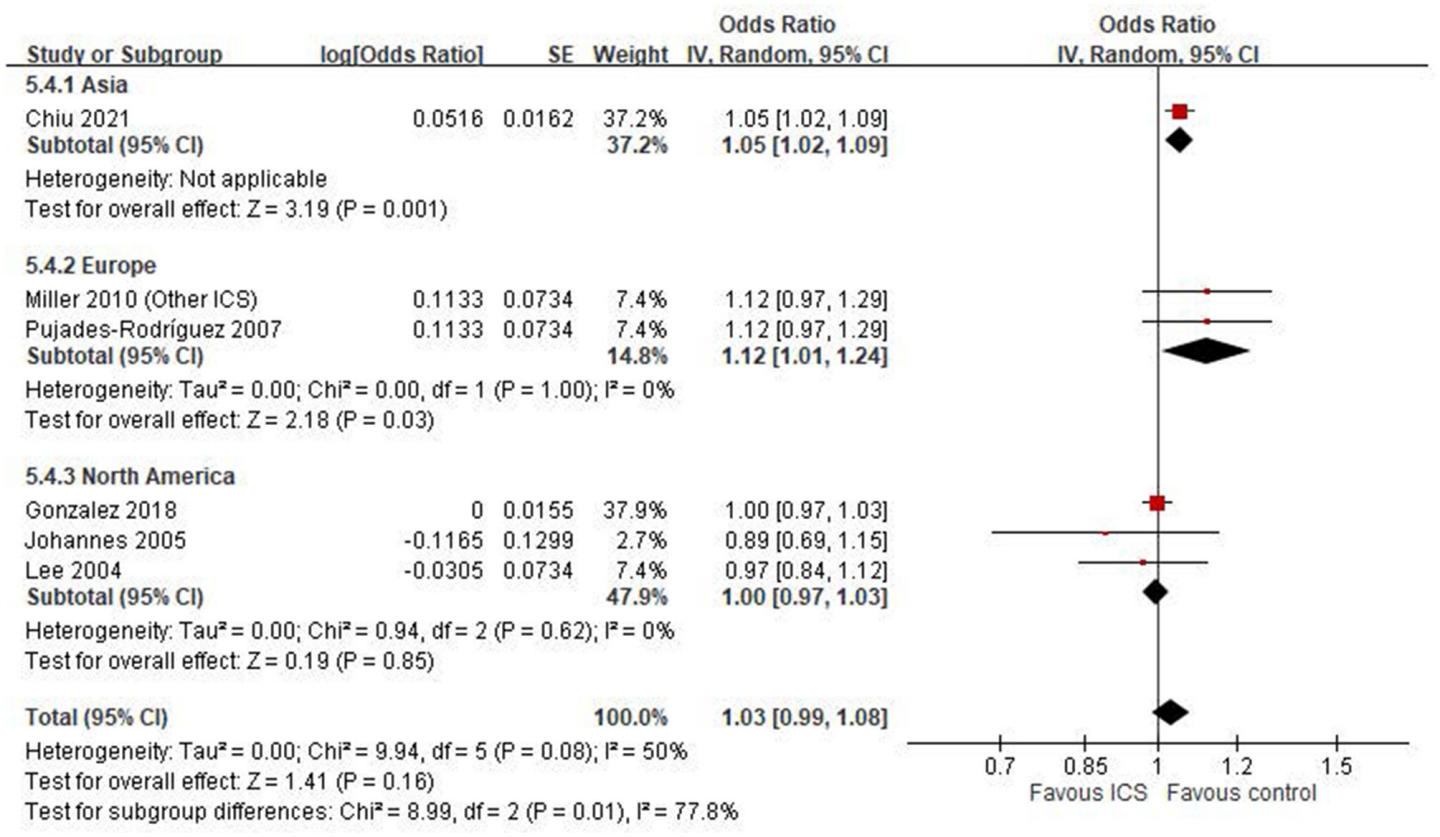
Subgroup analysis by geographic region of included studies.

A subgroup analysis based on study region revealed notable differences in the association between ICS use and fracture or osteoporosis risk (Figure 5). In the Asian subgroup, represented by a single large study (20), ICS use was significantly associated with an increased risk (OR: 1.05, 95% CI: 1.02–1.09, *P* = 0.001). In contrast, the pooled estimate from European studies (21, 24) also demonstrated a statistically significant increase in risk (OR: 1.12, 95% CI: 1.01–1.24, *P* = 0.03), with no observed heterogeneity (*I*^2^ = 0%). Conversely, studies conducted in North America (18, 22, 23) showed no significant association (OR: 1.00, 95% CI: 0.97–1.03, *P* = 0.85). Between-region heterogeneity was statistically significant (Ch*i*^2^ = 8.99, d*f* = 2, *P* = 0.01; *I*^2^ = 77.8%).

### Meta-regression analysis

**Figure 6:**
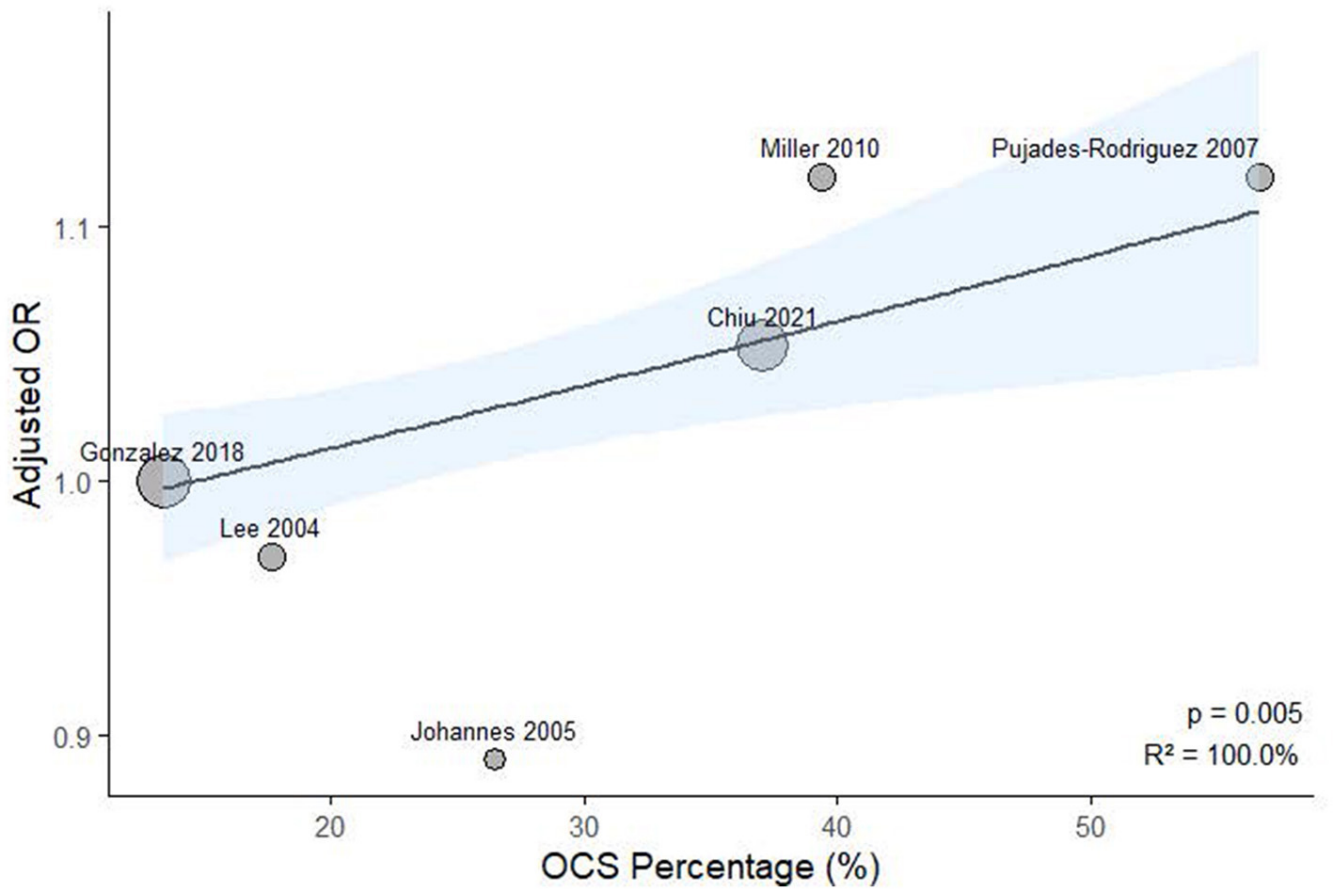
Meta-regression analysis examining the association between study-level oral corticosteroid exposure prevalence and ICS-related fracture or osteoporosis risk.

**Figure 7:**
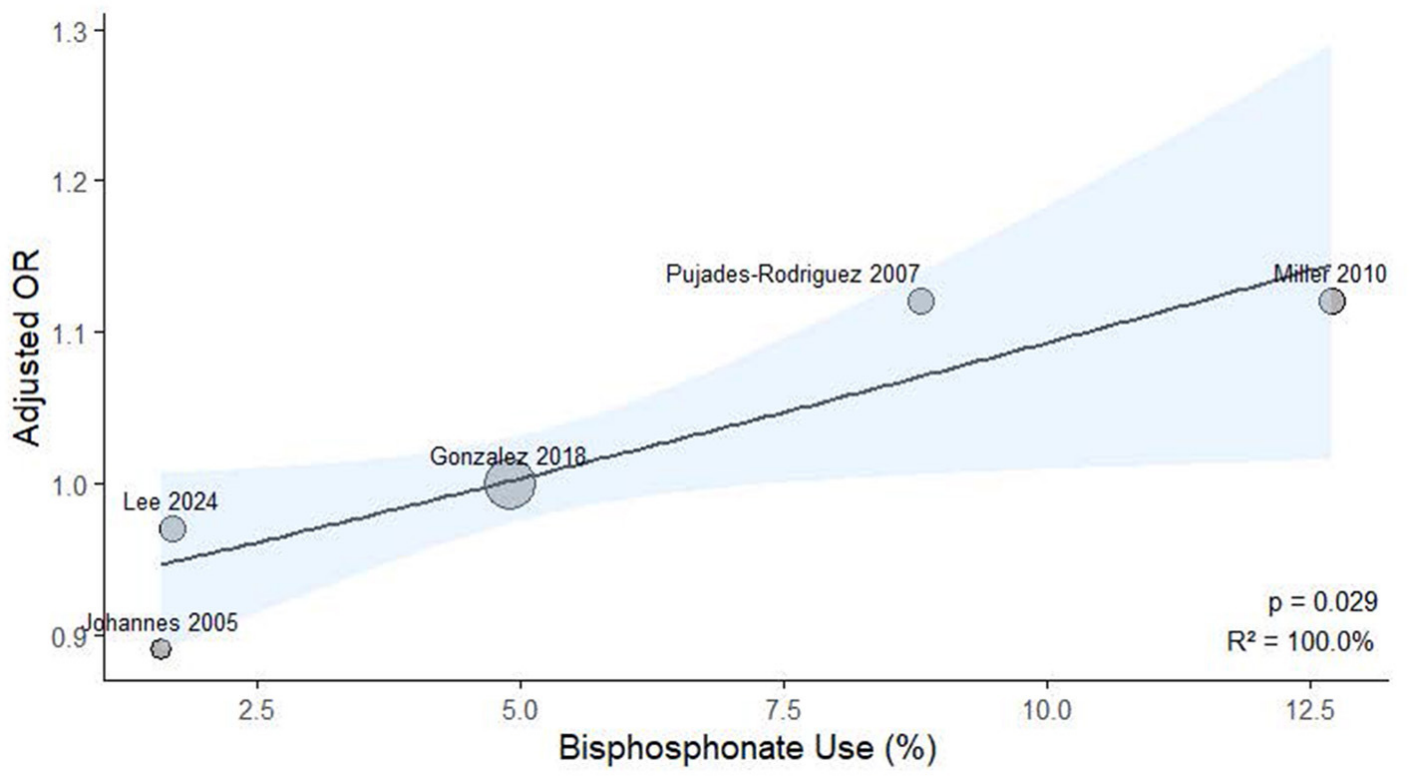
Meta-regression analysis examining the association between study-level bisphosphonate exposure prevalence and ICS-related fracture or osteoporosis risk.

To explore potential sources of heterogeneity, meta-regression analysis was performed using study-level covariates, including the proportion of patients receiving OCS and bisphosphonates. As illustrated in Figure 6, a significant positive association was observed between OCS prevalence and effect size (*p* = 0.005, *R*^2^ = 100.0%). Similarly, bisphosphonate use was also significantly associated with the effect estimates (*p* = 0.029, *R*^2^ = 100.0%), as shown in Figure 7.

### Sensitivity analysis

To evaluate the robustness of the findings, a sensitivity analysis was conducted by excluding Gonzalez et al. (18), which had a very large sample size and a near-null effect estimate. As shown in Supplementary Figure S1, the exclusion led to a slightly higher pooled odds ratio of 1.05 (95% CI: 1.01–1.10, *P* = 0.01), while heterogeneity was markedly reduced (*I*^2^ = 8%). The removal of other studies resulted in minor fluctuations in the effect estimate but did not materially alter the conclusions.

## Discussion

Our systematic review examined the impact of ICS on osteoporosis or fractures in patients with COPD. It did not find a statistically significant association between ICS use and osteoporosis or fractures in these patients. Our meta-analyses included both case-control and cohort studies, which inherently differ in study design and susceptibility to bias. The pooled estimate from six case-control studies showed no statistically significant association between ICS use and osteoporosis or fracture risk (OR = 1.03, 95% CI: 0.99–1.08; *I*^2^ = 50%), whereas the two cohort studies also showed no significant association (HR = 0.95, 95% CI: 0.67–1.33) but with high heterogeneity (*I*^2^ = 86%), indicating substantial variability in study populations, exposure definitions, and follow-up durations. In subgroup analyses, we found differences in effect estimates depending on outcome definitions. Only one study assessing osteoporosis specifically reported a significant association with ICS use (OR = 1.05), while other studies evaluating general fracture outcomes showed mixed results. Moreover, the geographic region appeared to influence the results. Asian and European studies showed a statistically significant increased risk associated with ICS, while North American studies reported null associations. This regional variation may reflect differences in clinical practice, population characteristics, healthcare systems, or confounding control. These methodological and contextual differences likely contributed to the observed heterogeneity and should be considered when interpreting the overall findings.

A previous random-effects meta-analysis that included patients with asthma or COPD assessed the long-term effects of ICS use on bone mineral density. It concluded that prolonged use of ICSs did not significantly alter bone mineral density in these groups (27). Another systematic review and meta-analysis also explored the impact of ICS on fracture risk, bone mineral density, and bone markers in patients with asthma and COPD, and it indicated an increased fracture risk (28).

Considering the distinct nature of COPD and asthma and the fact that COPD patients tend to have more comorbidities, such as cataracts, osteoporosis, coronary artery disease, pneumonia, and airway infections, the results of ICS use might differ between the two groups. In contrast, asthma patients typically exhibit fewer comorbidities, likely due to a younger age profile (29). Therefore, meta-analyses that include both asthma and COPD patients may not accurately represent the effects of ICS on individuals with COPD alone.

A meta-analysis sought to assess the advantages and risks of adding ICS treatment for patients with severe or very severe COPD. The analysis incorporated data from 9 randomized controlled trials and demonstrated that combining an ICS with a LABA reduced exacerbation rates but heightened the risks of pneumonia and oral candidiasis when compared to monotherapy with long-acting bronchodilators. Moreover, the incorporation of ICS markedly improved patient-perceived health and wellbeing (30). However, this study did not explore the potential effects on osteoporosis and fractures.

Previous systematic reviews have indicated that ICSs do not increase fracture risk in patients with COPD. However, these findings are subject to debate due to the limited number and early publication dates of the articles included (31–34).

A systematic review and meta-analysis of 44 randomized controlled trials, involving 87,594 patients, was conducted to determine the impact of inhaled corticosteroids on fracture risk in COPD patients. The analysis revealed that inhaled therapies containing ICSs, such as ICS/LABA combinations and triple therapy, were significantly associated with an increased risk of fractures in COPD patients compared to inhaled treatments without ICSs (35). The analysis only included randomized controlled trials (RCTs) that reported fracture event data. Trials that did not report fracture events, as well as non-randomized controlled trials such as observational studies, case series, and reviews, were excluded. Additionally, the study did not address the outcome of osteoporosis nor consider the use of oral corticosteroids. Simultaneously, another study analyzed parallel-group randomized controlled trials (RCTs) comparing ICS with non-ICS therapies in individuals with COPD, specifically looking at reported adverse events including fractures or osteoporosis. This meta-analysis included 61,380 participants from 26 RCTs and found that ICS did not increase the risk of fractures or osteoporosis (36).

The study faced multiple limitations. Primarily, the meta-analysis included only RCTs, omitting observational studies which could offer valuable information on the long-term impacts of ICS therapy in patients with COPD. Moreover, the majority of these RCTs concentrated on evaluating respiratory outcomes and mortality rates, and did not implement precise methodologies to accurately identify incidents of fractures or osteoporosis. This lack of specificity could significantly heighten the possibility of misclassification or underdiagnosis of these conditions. Additionally, the mean duration of therapy across the included trials was ~1–2 years, a timeframe that may be insufficient to fully capture the side effects associated with ICS, such as fractures and osteoporosis.

Our study included both cohort and case-control studies that reported on the outcomes of osteoporosis or fractures, observing participants for a period exceeding 4 years. Additionally, we considered the use of oral steroids in our analysis. Despite this comprehensive approach, we were unable to demonstrate a statistically significant increase in osteoporosis or fractures among individuals with COPD who received ICS compared to those with COPD who were not exposed to ICS. However, in meta-regression, we did find study-level oral corticosteroid prevalence revealed a significant positive association, indicating that higher background exposure to oral corticosteroids was correlated with a greater risk of osteoporosis or fractures among ICS users.

Our systematic review focused on cohort or case-control studies that assessed the impact of ICS on osteoporosis or fractures in patients with COPD. We found no consistent evidence of a significant adverse relationship between ICS use and specific sites such as the lumbar spine or femur. Additionally, there was insufficient data to establish any dose-response relationship or to evaluate potential differences between various ICS compounds. Our results should be viewed in light of other recent publications. Two systematic reviews specifically investigated fractures or osteoporosis in ICS users with COPD: one found that ICS exposure did not increase the risk of fractures or osteoporosis (36), while other recent meta-analyses have reported a statistically significant increase in the risk of fractures associated with ICS use in these patients (35).

Our research revealed that exposure to ICS did not elevate the risk of fractures or osteoporosis in COPD patients. A meta-regression using continuous study-level oral corticosteroids prevalence identified a statistically significant trend (*p* = 0.005; *R*^2^ = 100%), indicating that higher oral corticosteroids exposure was associated with stronger effect estimates. Additionally, a meta-regression using study-level bisphosphonate use as a covariate revealed a significant positive association with ICS-related risk (*p* = 0.029; *R*^2^ = 100%). These results imply that ICS-associated fracture risk may be influenced by concomitant oral corticosteroid use, and that higher bisphosphonate use may indicate a study population with pre-existing skeletal fragility. We concentrated specifically on the use of ICS in patients with COPD, utilizing cohort or case-control studies, which allowed for longer observation periods compared to randomized controlled trials. To ensure the accuracy of our findings, we employed stringent selection criteria designed to comprehensively include COPD patients and exclude those with asthma, and we conducted our analysis over extended periods in these observational studies.

Oral corticosteroids are commonly used to manage acute exacerbations in COPD. Patients should be counseled to use the lowest effective dose that adequately controls their symptoms and minimizes future risk of osteoporosis, thereby reducing the likelihood of bone-related side effects. Screening for bone density and managing osteoporosis should be prioritized in populations at high risk in patients with COPD. Physicians need to be particularly vigilant about the risk of fractures in patients with COPD using oral corticosteroids over the long term.

Considering the high prevalence of COPD, the often necessary use of systemic steroid and ICS therapy, and the associated increased risks of osteoporosis and fractures, it is essential to develop COPD-specific guidelines for bone protection. These guidelines would serve to inform and educate both clinicians and patients, with the goal of reducing the incidence of preventable osteoporotic fractures.

## Limitations

We fully acknowledge that several critical variables, such as cumulative ICS dosage, treatment duration, baseline COPD severity, smoking status, physical activity level, and history of prior fractures were not analyzed in our review. This is primarily due to the lack of consistently reported data across the included studies, which prevented us from conducting further stratified or adjusted analyses.

We acknowledge that the relatively small number of included studies may limit the generalizability of our findings. We emphasize the need for further high-quality studies in diverse populations to validate our conclusions. To better understand the observed heterogeneity, we conducted subgroup analyses based on outcome definitions and geographic region, as well as a meta-regression using oral corticosteroid and bisphosphonate prevalence as a continuous covariate. These additional analyses revealed that differences in clinical definitions, geographic context, and background oral corticosteroid exposure may have contributed significantly to inter-study variability. We have expanded our discussion on the inherent limitations of observational research, particularly the susceptibility to residual confounding. Although we used the NOS to assess study quality, we recognize that unmeasured or uncontrolled confounders (e.g., comorbidities, medication adherence) may still influence the results. As noted, most included studies did not differentiate between ICS monotherapy and ICS combined with oral corticosteroid. While this is a significant limitation, we addressed it analytically by performing a study-level meta-regression using oral corticosteroid prevalence, which revealed a significant trend, suggesting that higher oral corticosteroid exposure may amplify the ICS-associated risk.

## Conclusion

Although ICS did not significantly affect the risk of osteoporosis or fractures in our study, these conditions are common comorbidities in patients with COPD. Methodological differences among the included studies, such as study design (cohort vs. case-control), definitions of outcomes, and variations in oral corticosteroid use may influence the interpretation of the results. These differences contribute to clinical and methodological heterogeneity, which may affect effect estimates and limit the comparability across studies.

## Data Availability

The original contributions presented in the study are included in the article/Supplementary material, further inquiries can be directed to the corresponding author.
