# Supplementary material for: Evaluating inhaled corticosteroids' impact on osteoporosis and fracture risk in COPD patients: a real-world evidence-based systematic review and meta-analysis
Source: Front Med (Lausanne). 2025 Jun 6;12:1503475. doi: 10.3389/fmed.2025.1503475 (PMC12178898; doi:10.3389/fmed.2025.1503475)
Supplement: Supplementary file 1 [file Table_1.docx]

Table S1. Search strategy

**Database: PubMed**

| No | Query | Results |
| --- | --- | --- |
| 1 | Pulmonary Disease, Chronic Obstructive | 106,770 |
| 2 | (COPD) OR (Chronic Obstructive Lung Disease*) OR (Chronic Obstructive Pulmonary Disease*) OR (Chronic Obstructive Airway Disease*) OR (Chronic Obstructive Pulmonary Disease*) OR (chronic obstructive bronchopulmonary disease*) OR (chronic obstructive respiratory disease*) | 127,461 |
| 3 | #1 OR #2 | 127,461 |
| 4 | (Inhalation) OR (Administration, Inhalation) | 195,260 |
| 5 | (Inhal*) OR (Inspiration) OR (aerosol) | 353,343 |
| 6 | Nebulizers and Vaporizers | 13,406 |
| 7 | (Vaporizer*) OR (Inhalers) OR (Inhaler) OR (Inhalator*) OR (Nebulizer*) OR (Atomizer*) | 198,445 |
| 8 | #4 OR #5 OR #6 OR #7 | 357,720 |
| 9 | Adrenal Cortex Hormones | 315,164 |
| 10 | (Corticosteroid*) OR (Corticoid*) | 140,085 |
| 11 | Budesonide | 7,797 |
| 12 | (Budesonide*) OR (Pulmicort) | 7,823 |
| 13 | Fluticasone | 5,413 |
| 14 | (Fluticasone*) OR (Flixonase) OR (Flixotide) OR (Flovent) OR (Flonase) OR (Flovent) OR (Cutivate) | 5,440 |
| 15 | Beclomethasone | 4,160 |
| 16 | (Ciclesonide) OR (alvesco) OR (aservo) OR (omnaris) OR (zetonna) OR ('zetonna nasal aerosol') OR (ciclesonide*) | 505 |
| 17 | (Flunisolide) OR (Flunisolide*) | 389 |
| 18 | Mometasone Furoate | 1,324 |
| 19 | Mometasone* | 1,499 |
| 20 | #9 OR #10 OR #11 OR #12 OR #13 OR #14 OR #15 OR #16 OR #17 OR #18 OR #19 | 407,247 |
| 21 | #8 AND #20 | 25,290 |
| 22 | #3 AND #21 | 5,154 |
| 23 | (cohort studies[mh] OR cohort*[tw] OR controlled clinical trial[pt] OR case-control studies[mh] OR (case*[tw] AND (control*[tw] OR crossover[tw] OR cross-over[tw] OR comparison*[tw])) "control group"[tw] OR "control groups"[tw] OR risk*[tw] OR incidence*[tw] OR (epidemiologic methods[mh:noexp] )) | 4,746,206 |
| 24 | #22 AND #23 | 1,904 |

**Database: Embase**

| No. | Query | Results |
| --- | --- | --- |
| 1 | 'chronic obstructive lung disease'/exp OR 'chronic obstructive lung disease' | 202,632 |
| 2 | ((((((copd OR chronic) AND obstructive AND lung AND disease* OR chronic) AND obstructive AND pulmonary AND disease* OR chronic) AND obstructive AND airway AND disease* OR chronic) AND obstructive AND pulmonary AND disease* OR chronic) AND obstructive AND bronchopulmonary AND disease* OR chronic) AND obstructive AND respiratory AND disease* | 113,550 |
| 3 | #1 OR #2 | 213,050 |
| 4 | 'inhalational drug administration'/exp | 52,540 |
| 5 | inhal* OR inspiration OR aerosol OR vaporizer* OR inhalers OR inhaler OR inhalator* OR nebulizer* OR atomizer* | 412,881 |
| 6 | 'corticosteroid'/exp | 1,288,830 |
| 7 | corticosteroid* OR corticoid* | 441,239 |
| 8 | 'budesonide'/exp | 27,304 |
| 9 | budesonide OR pulmicort | 30,774 |
| 10 | 'fluticasone'/exp | 10,188 |
| 11 | flixonase OR flixotide OR flonase OR flovent OR cutivate | 1,560 |
| 12 | 'beclometasone'/exp | 8,357 |
| 13 | beclomethasone* | 10,507 |
| 14 | 'ciclesonide'/exp | 2,179 |
| 15 | alvesco OR aservo OR omnaris OR zetonna OR 'zetonna nasal aerosol' OR ciclesonide* | 2,250 |
| 16 | 'flunisolide'/exp | 2,505 |
| 17 | flunisolide* | 2,545 |
| 18 | 'mometasone furoate'/exp | 7,006 |
| 19 | mometasone* | 7,378 |
| 20 | #4 OR #5 | 412,881 |
| 21 | #6 OR #7 OR #8 OR #9 OR #10 OR #11 OR #12 OR #13 OR #14 OR #15 OR #16 OR #17 OR #18 OR #19 | 1,325,678 |
| 22 | #20 AND #21 | 75,414 |
| 23 | 'osteoporosis'/exp | 170,183 |
| 24 | (osteoporos* OR bone) AND densit* | 199,174 |
| 25 | 'corticosteroid induced osteoporosis'/exp | 2,182 |
| 26 | 'fracture'/exp | 425,769 |
| 27 | fracture* | 540,367 |
| 28 | (skeletal OR bone) AND health | 377,129 |
| 29 | #23 OR #24 OR #25 OR #26 OR #27 OR #28 | 1,032,149 |
| 30 | #3 AND #22 AND #29 | 880 |

**Database: Scopus**

Strategy:

( TITLE-ABS-KEY ( copd ) AND TITLE-ABS-KEY ( inhalation ) AND TITLE-ABS-KEY ( corticosteroid OR budesonide OR fluticasone OR beclomethasone OR ciclesonide OR flunisolide OR mometasone ) AND TITLE-ABS-KEY ( osteoporosis OR fractures OR ( bone AND health ) OR ( bone AND density ) OR ( skeletal AND health ) ) )

**Database: Web of Science**

Strategy:

(Pulmonary Disease, Chronic Obstructive OR COPD OR Chronic Obstructive Lung Disease* OR Chronic Obstructive Pulmonary Disease* OR Chronic Obstructive Airway Disease* OR Chronic Obstructive Pulmonary Disease* OR chronic obstructive bronchopulmonary disease* OR chronic obstructive respiratory disease*) AND Inhalation AND (Adrenal Cortex Hormones OR Corticosteroid* OR Corticoid* OR Budesonide OR Fluticasone OR Beclomethasone OR Ciclesonide OR Flunisolide OR Mometasone)

Table S2. Studies excluded after assessment of full-text articles

| **SN** | **Reasons for exclusion** | **Authors** | **Year** | **Title** | **Journal** | **Vol** | **No** | **Page** |
| --- | --- | --- | --- | --- | --- | --- | --- | --- |
| 1 | Duplicated studies | O. Johnell; R. Pauwels; C. G. Löfdahl; L. A. Laitininen; D. S. Postma; N. B. Pride; S. V. Ohlsson | 2002 | Bone mineral density in patients with chronic obstructive pulmonary disease treated with budesonide Turbuhaler® | European Respiratory Journal | 19 | 6 | 1058-1063 |
| 2 | Duplicated studies | R. Meyer | 2018 | COPD: More fractures with inhaled glucocorticoids | Deutsches Arzteblatt International | 115 | 8 | A341 |
| 3 | Duplicated studies | P. C. Lu; Y. H. Yang; S. E. Guo; T. M. Yang | 2017 | Factors associated with osteoporosis in patients with chronic obstructive pulmonary disease—a nationwide retrospective study | Osteoporosis International | 28 | 1 | 359-367 |
| 4 | Duplicated studies | S. Gonnelli; C. Caffarelli; S. Maggi; S. Rossi; P. Siviero; G. Crepaldi; R. Nuti | 2010 | In copd inhaled glucocorticoids, but not B2-agonist are associated with vertebral fracture risk | Journal of Bone and Mineral Research | 25 |  | S118 |
| 5 | Duplicated studies | M. Pujades-Rodríguez; C. J. P. Smith; R. B. Hubbard | 2007 | Inhaled corticosteroids and the risk of fracture in chronic obstructive pulmonary disease | QJM | 100 | 8 | 509-517 |
| 6 | Randomized controlled trial | O. Johnell; R. Pauwels; C. G. Löfdahl; L. A. Laitinen; D. S. Postma; N. B. Pride; S. V. Ohlsson | 2002 | Bone mineral density in patients with chronic obstructive pulmonary disease treated with budesonide Turbuhaler | Eur Respir J | 19 | 6 | 1058-63 |
| 7 | Randomized controlled trial | A. Struijs; H. Mulder | 1997 | The effects of inhaled glucocorticoids on bone mass and biochemical markers of bone homeostasis: A 1-year study of beclomethasone versus budesonide | Netherlands Journal of Medicine | 50 | 6 | 233-237 |
| 8 | Cross-sectional studies | C. E. McEvoy; K. E. Ensrud; E. Bender; H. K. Genant; W. Yu; J. M. Griffith; D. E. Niewoehner | 1998 | Association between corticosteroid use and vertebral fractures in older men with chronic obstructive pulmonary disease | American Journal of Respiratory and Critical Care Medicine | 157 | 3 I | 704-709 |
| 9 | Cross-sectional studies | S. Gonnelli; C. Caffarelli; S. Maggi; G. Guglielmi; P. Siviero; S. Rossi; G. Crepaldi; R. Nuti | 2010 | Effect of inhaled glucocorticoids and beta(2) agonists on vertebral fracture risk in COPD patients: the EOLO study | Calcif Tissue Int | 87 | 2 | 137-43 |
| 10 | Cross-sectional studies | V. J. Guerrero Sánchez; O. R. Rincón; U. C. Uscategui; A. D. Alfonso; C. M. Carreño | 2022 | FRAGILITY FRACTURES AMONG PATIENTS WITH CHRONIC OBSTRUCTIVE PULMONARY DISEASE (COPD) EXPOSED AND UNEXPOSED TO GLUCOCORTICOIDS | Aging Clinical and Experimental Research | 34 |  | S417 |
| 11 | Cross-sectional studies | B. J. Zarowitz; T. O'Shea | 2013 | Inhaled corticosteroid (ICS) use in nursing home (NH) residents with copd | Value in Health | 16 | 3 | A231 |
| 12 | Cross-sectional studies | A. V. Gonzalez; J. Coulombe; P. Ernst; S. Suissa | 2018 | Long-term Use of Inhaled Corticosteroids in COPD and the Risk of Fracture | Chest | 153 | 2 | 321-328 |
| 13 | Studies with mixed groups of participants (asthma/COPD) | A. Langhammer; S. Forsmo; S. Lilleeng; R. Johnsen; L. Bjermer | 2007 | Effect of inhaled corticosteroids on forearm bone mineral density: the HUNT study, Norway | Respir Med | 101 | 8 | 1744-52 |
| 14 | Studies with mixed groups of participants (asthma/COPD) | B. C. Ng; W. D. Leslie; K. M. Johnson; J. M. FitzGerald; M. Sadatsafavi; W. Chen | 2020 | Effects of long-term inhaled corticosteroid treatment on fragility fractures in older women: the Manitoba BMD registry study | Osteoporosis International | 31 | 6 | 1155-1162 |
| 15 | Studies with mixed groups of participants (asthma/COPD) | P. Vestergaard; L. Rejnmark; L. Mosekilde | 2007 | Fracture risk in patients with chronic lung diseases treated with bronchodilator drugs and inhaled and oral corticosteroids | Chest | 132 | 5 | 1599-1607 |
| 16 | Studies with mixed groups of participants (asthma/COPD) | R. B. Hubbard; C. J. P. Smith; L. Smeeth; T. W. Harrison; A. E. Tattersfield | 2002 | Inhaled corticosteroids and hip fracture: A population-based case-control study | American Journal of Respiratory and Critical Care Medicine | 166 | 12 I | 1563-1566 |
| 17 | Studies with mixed groups of participants (asthma/COPD) | C. H. Tsai; L. Y. Liao; C. L. Lin; W. S. Chung | 2018 | Inhaled corticosteroids and the risks of low-energy fractures in patients with chronic airway diseases: A propensity score matched study | Clinical Respiratory Journal | 12 | 5 | 1830-1837 |
| 18 | Studies with mixed groups of participants (asthma/COPD) | F. de Vries; T. P. van Staa; M. S. Bracke; C. Cooper; H. G. Leufkens; J. W. Lammers | 2005 | Severity of obstructive airway disease and risk of osteoporotic fracture | Eur Respir J | 25 | 5 | 879-84 |
| 19 | Studies with mixed groups of participants (asthma/COPD) | A. Grosso; L. Cazzoletti; F. Albicini; E. Gini; E. Zanolin; V. Ronzoni; V. Conio; R. Di Domenica; A. G. Corsico; D. Jarvis; C. Janson; I. Cerveri | 2018 | Use of inhaled corticosteroids and the risk of osteoporosis: An international cohort study | European Respiratory Journal | 52 |  |  |
| 20 | Review articles | J. Steurer | 2018 | COPD: Years of high doses of inhaled steroids increase the risk of fracture | Praxis | 107 | 13 | 731-732 |
| 21 | Review articles |  | 2004 | ICS use elevates fracture risk in COPD patients | Formulary | 39 | 5 | 250 |
| 22 | Review articles | J. Y. Moon; D. D. Sin | 2019 | Inhaled corticosteroids and fractures in chronic obstructive pulmonary disease: Current understanding and recommendations | Current Opinion in Pulmonary Medicine | 25 | 2 | 165-172 |
| 23 | Review articles | N. Putcha; M. B. Drummond | 2012 | Inhaled corticosteroids for people with chronic obstructive pulmonary disease are associated with an increased risk of fracture | Evidence-Based Medicine | 17 | 3 | 90-91 |
| 24 | Review articles |  | 2011 | Inhaled corticosteroids increase fracture risk in COPD | Drug and Therapeutics Bulletin | 49 | 8 | 88 |
| 25 | Review articles | T. L. Petty | 2004 | The risk of osteoporosis in patients with COPD | Consultant | 44 | 4 | 560 |
| 26 | Both groups used ICS | Q. Huangfu; M. Li; L. Xiao; H. Tao; W. Wang; X. Fei | 2018 | Effect of inhaled glucocorticoids on chronic obstructive pulmonary disease in male patients with osteoporosis | Farmacia | 66 | 1 | 70-77 |
| 27 | Both groups used ICS | P. C. Lu; Y. H. Yang; S. E. Guo; T. M. Yang | 2017 | Factors associated with osteoporosis in patients with chronic obstructive pulmonary disease-a nationwide retrospective study | Osteoporos Int | 28 | 1 | 359-367 |
| 28 | Conference abstracts | H. C. Kuo | 2015 | Dose response protective effect of inhaled corticosteroid on osteoporosis in patients with chronic obstructive pulmonary disease | Respirology | 20 |  | 32 |
| 29 | Conference abstracts | S. Suissa; J. Coulombe; P. Ernst | 2016 | Long-term inhaled corticosteroid use in COPD and the risk of fracture in men and women | European Respiratory Journal | 48 |  |  |
| 30 | Both groups used ICS | W. D. Pace; E. Callen; G. Gaona-Villarreal; A. Shaikh; B. P. Yawn | 2025 | Adverse Outcomes Associated With Inhaled Corticosteroid Use in Individuals With Chronic Obstructive Pulmonary Disease | Ann Fam Med | 23 | 2 | 127-135 |


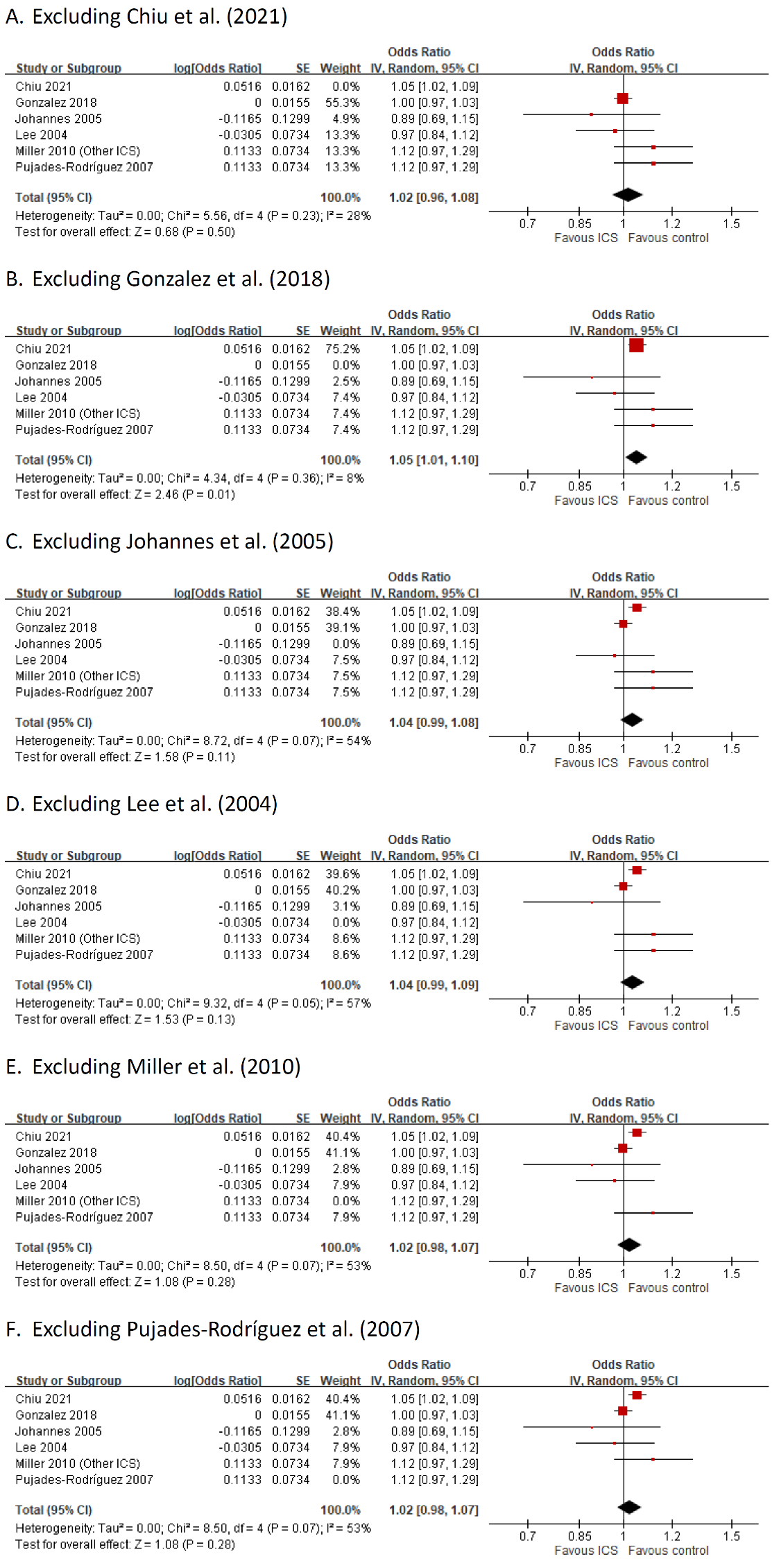


Figure S1. Leave-one-out sensitivity analysis of case-control studies assessing the association between inhaled corticosteroid (ICS) use and risk of fracture or osteoporosis.
